# High-performance CT features supporting accurate pre-operative tumor staging in colon cancer

**DOI:** 10.3389/fonc.2025.1549075

**Published:** 2025-02-10

**Authors:** Jianhua Yuan, Cangzheng Jin, Jianrong Si, Baobao Liu, Xiaohan Si, Jianzhi Chen

**Affiliations:** ^1^ Department of Radiology, Guangdong Provincial Hospital of Integrated Traditional Chinese and Western Medicine, Foshan, China; ^2^ Department of Radiology, The Sixth Affiliated Hospital, School of Medicine, South China University of Technology, Foshan, China; ^3^ Department of Pathology, Guangdong Provincial Hospital of Integrated Traditional Chinese and Western Medicine (affiliated to Guangzhou Kingmed Diagnostics Group Co. Ltd.), Foshan, China; ^4^ Ecosystem Change and Population Health Research Group, School of Public Health and Social Work, Queensland University of Technology, Brisbane, QLD, Australia; ^5^ Department of Pathology, The Sixth Affiliated Hospital, School of Medicine, South China University of Technology, Foshan, China

**Keywords:** colon cancer, CT feature, serosal side, pathological T staging, clinical T staging

## Abstract

**Background and aims:**

Accurate pre-treatment tumor staging is essential for treatment decision-making. Multi-slice spiral computed tomography (CT) is currently the standard method for pre-operative clinical tumor staging, but accurately applying the CT findings in tumor staging remains a challenge due to the poor pathological understanding of the CT signs. We aimed to pathologically interpret the key CT findings in order to identify reliable markers for pre-treatment staging of colon cancer.

**Patients and methods:**

The following CT features from 136 colon adenocarcinomas were analyzed: colon wall pliability, outline contour, pericolic fat attenuations and vascularity, tumor fusion with adjacent organs, ascites, tumor size, and relevance between tumor and retroperitoneal fascia. These CT features were corroborated with histopathological findings. The diagnostic performance of these was further analyzed.

**Results:**

Based on the postoperative pathological tumor staging (pT), 136 colon adenocarcinomas were classified into four stages: pT1 (n = 5), pT2 (n = 7), pT3 (n = 96), and pT4 (n = 28). Key findings include the following: (1) soft colon wall is a characteristic of the pT1 tumors, whereas stiff colon wall is a characteristic of the pT2~pT4 tumors; pathologically, stiff colon wall reflects the infiltration of tumor cells with desmoplastic reaction (DR) in muscularis propria; (2) small protuberances may help exclude the pT2 tumors. Histopathologically, small protuberances in pT2 cancer represent the pure DR on the surface of lamina propria, whereas the small protuberances in pT3~pT4 cancers represent the sub-serosal or extra-serosal cancer cell foci enwrapped by DR; (3) the presence of large protuberances, extensive reticulonodular fat stranding, and fusion with surrounding organs and ascites are diagnostic of pT4 tumors; and (4) the presence of extra-fascial nodules/streaks on CT scan could accurately diagnose the ascending/descending colon cancer of pT4 stage. Histologically, the presence of the above five CT features (large protuberances, extensive reticulonodular fat stranding, fusion with surrounding organs, ascites, and extra-fascial nodules/streaks) reflect the farther and deeper infiltration of tumor cells in serosa or retroperitoneal fascia involvement.

**Conclusion:**

Our studies have identified multiple CT features that are practically useful in identifying and differentiating different stages of colon cancer prior to surgical procedures. These high-performance markers will provide valuable insights to the clinicians in making appropriate decisions in the management of patients with colon cancer.

## 1 Introduction

Neoadjuvant chemotherapy is a widely accepted therapeutic approach for colorectal cancer (CRC), but its success heavily relies on accurate pre-treatment Tumor-Node-Metastasis (TNM) staging (1–4). Currently, multi-slice spiral computed tomography (CT) is the standard method for pre-operative clinical T staging (4–7), as this modality has several advantages such as the ability to clearly depict the serosal fat layer and intra-abdominal fat (presenting as lower attenuation with negative CT values), whereas the smooth outer contour of normal colon (i.e., the serosal surface of muscularis propria) can be clearly revealed in the fat background. As such, the serosal profile of colon cancer and other abnormalities in the surrounding tissues may standout on CT scan. However, the rate of correlation between the imaging findings from the CT-based clinical tumor staging and the postoperative pathological tumor staging (pT) is relatively low, ranging from 30% to 76% (8–14). This disparity is primarily due to several shortcomings of CT imaging in CRC diagnosis, including the inability to precisely recognize the level of tumor penetration in submucosa, muscularis propria and serosal layer, and inability to distinguish cancer cells from accompanying desmoplastic reaction (DR) and inflammatory reaction (IR) (15–21).

In this study, we aimed to better understand the histopathological basis of the CT findings at the serosal side of colon cancer. The data derived from this study will facilitate the identification of key CT features that can potentially improve the accuracy of clinical T staging and accurate diagnosis of colon cancer. To the best of our knowledge, our innovative study is the first of its type to corroborate the CT features of the colon cancer with underlying pathology in clinical T staging.

## 2 Materials and methods

### 2.1 Study subjects

A total of 136 colon adenocarcinoma lesions from 131 patients who underwent radical surgery for colon cancer at the Guangdong Provincial Hospital of Integrated Traditional Chinese and Western Medicine between January 2010 and May 2021 were included in this study. Patient information was obtained from the Picture Archiving and Communication System (PACS) and Hospital Information System (HIS). Only the patients fulfilled the following criteria were included: (1) had pre-operative colon CT examination; (2) segment of colon cancer was completely resected; (3) entire pathological specimens and records were available; and; (4) final pathological TNM staging was performed. Patients were excluded if they show the following conditions: (1) the size and site of the cancer focus were indeterminate on CT; (2) the segment of colon cancer was not resected completely; and (3) presence of abdominal comorbidities such as intussusception, intestinal perforation, and peritonitis. All patients undertook radical surgery for colon cancer within 3–10 days after CT examination. This study was approved by the Ethics Committee of Guangdong Provincial Hospital of Integrated Traditional Chinese and Western Medicine. Because this is a retrospective cross-sectional study, informed consents from patients were not required.

### 2.2 CT examination techniques and parameters

All patients fasted for at least 8 h prior to the CT examination. Stomach and small intestine were dilated by oral administration of a 2.5% mannitol solution, and colon was dilatated by using the same solution via enema. CT scanning was carried out using the Toshiba Activion 16-Slice and Siemens Somatom Definition 64-Slice Helical CT scanners with the following parameters: tube voltage of 120 kV, automatic mode of tube current modulation, slice thickness of 1 mm, and pitch of 0.75. Images of the sagittal, coronal, and axial planes were reconstructed with a slice thickness of 3 mm on a CT workstation. The scanning field ranged from the top of diaphragm to the sciatic nodules. In images from the plain scan, arterial and portal venous phases were acquired. For contrast CT scan, the non-ionic iodine-containing contrast agent (300 mg of iodine/mL) was intravenously injected at a dose of 1.5 mL/kg body weight and a flow rate of 3.0 mL/s. All images were analyzed by two experienced radiologists on a PACS workstation to reach a consensus.

### 2.3 Histopathological examination

The surgically resected specimen was fixed in 10% neutral buffered formalin, embedded in paraffin, cut into sections of 4μm thickness, and then processed for standard change to hematoxylin-eosin staining (H&E). All sections were analyzed by two experienced pathologists to reach a consensus.

### 2.4 Statistical analysis

All statistical analyses were conducted using SPSS 23.0 software. Analysis of variance (ANOVA) was used to compare the mean values of the maximum diameter of the tumors in different groups. *Chi*-square (*x* ^2^) test was used to compare the difference in constituent ratios between groups. In cases where one or more theoretical frequencies (T) were in the range of 1–5, and the sample size (n) was >40, a corrected *x* ^2^ value was used for analysis. In case the T was <1 or the sample size (n) was <40, the exact Fisher’s test (F-value) was used. A *p* < 0.05 was considered statistically significant. For convenience, in this study, all *Chi*-square tests and *Chi*-square tests with Yates’ correction for continuity will be used to report the statistics value and *p*-value, but, for Fisher’s exact test, only *p*-value is reported.

To assess the diagnostic performance of the CT features of the serosal side of colon cancer, sensitivity (Se), specificity (Sp), positive predictive value (PPV), negative predictive value (NPV), positive likelihood ratio (+LR), and negative likelihood ratio (−LR) were calculated.

## 3 Results

### 3.1 Number and location of colon cancers

A total of 136 colon adenocarcinoma lesions from 131 patients (80 men and 51 women, aged 28 to 87 years, mean age of 64.5 ± 13.2 years) were included in this study. The vast majority of the patients had only one tumor (128/131), one had two tumors (1/131), and two had three tumors (2/131). The most common location for these tumors were sigmoid colon (n = 64, 47.1%), transverse colon (n = 28, 20.6%), ascending colon (n = 25, 18.4%), descending colon (n = 17, 12.5%), and cecum (n = 2, 1.5%). According to eighth edition AJCC Cancer Staging Manual (22), five tumors were at pT1, seven at pT2, 96 at pT3, and 28 at pT4.

### 3.2 Analysis of key CT features of colon cancers

**Figure 1:**
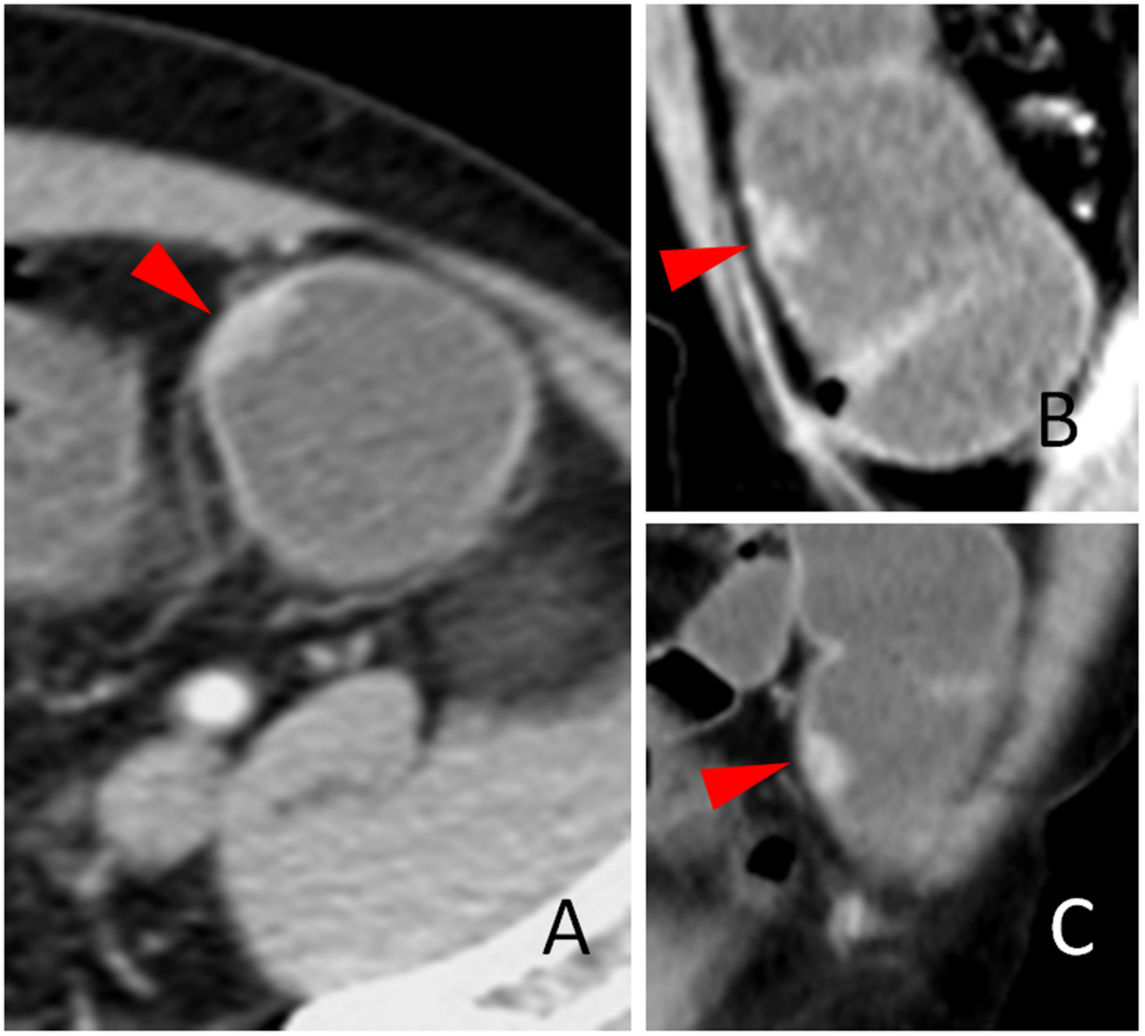
The soft wall sign. The transverse **(A)**, sagittal **(B)**, and coronal **(C)** sections of a colon adenocarcinoma at the middle sigmoid (red arrowheads), pT1N0Mx, in a 66-year-old man are shown. Arrows heads indicate soft colon wall, where the serosal side outline of the tumor exhibits a natural, compliant, and smooth characteristic and can be expanded. The outline is the same of the normal colon.

**Figure 2:**
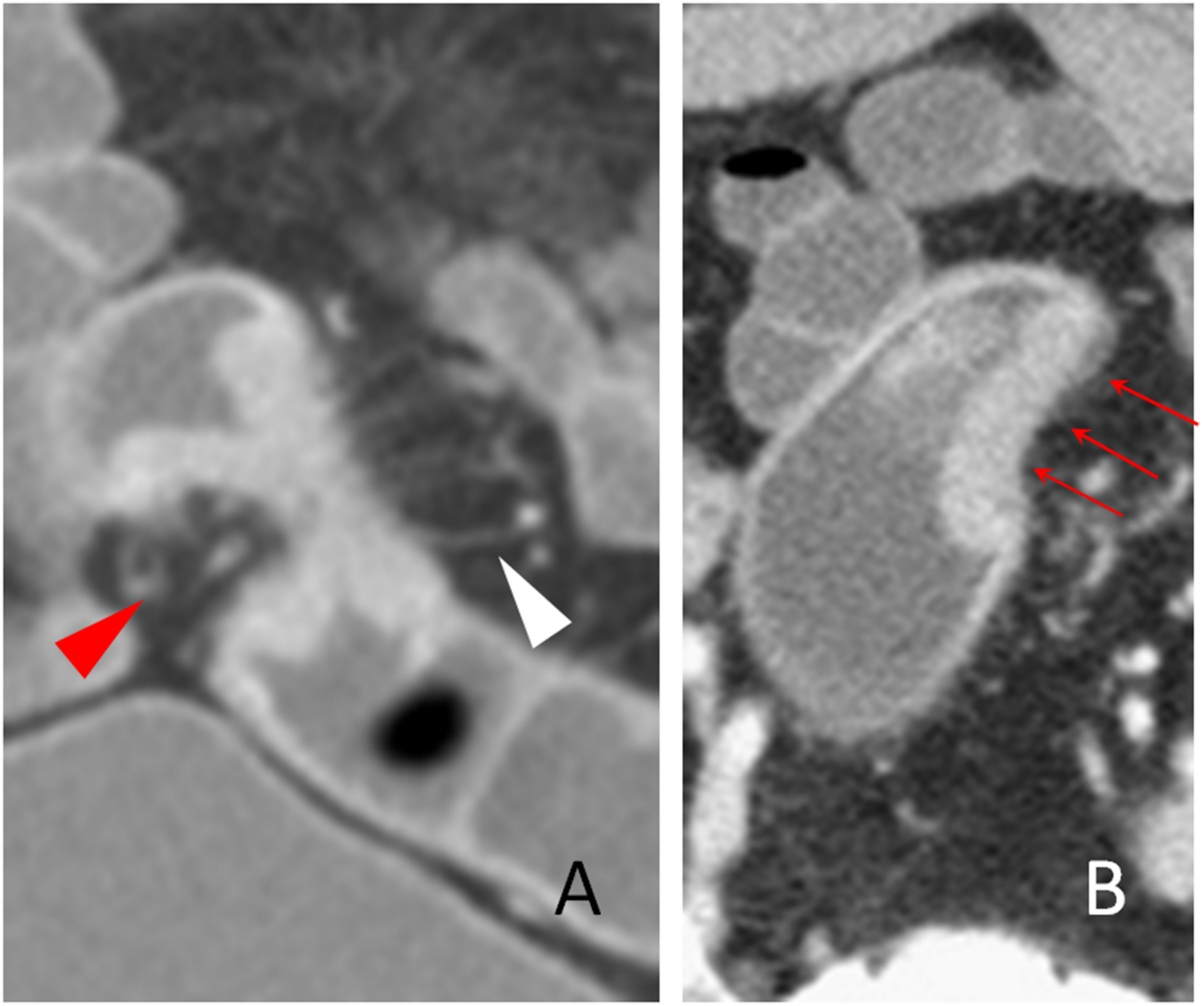
The stiff wall sign. The coronal **(A)** and transverse **(B)** sections of a distal sigmoid colon adenocarcinoma, pT2N0Mx, in a 63-year-old man are shown. The stiff colon wall (red arrow head) and increased pericolic blood vessels (white arrow head) are shown. The smooth but stiff serosal side outline of the tumor is shown (**B**, red arrows). Note that the stiff colon wall of the serosal side outline cannot be expanded and is concave toward the lumen.

**Figure 3:**
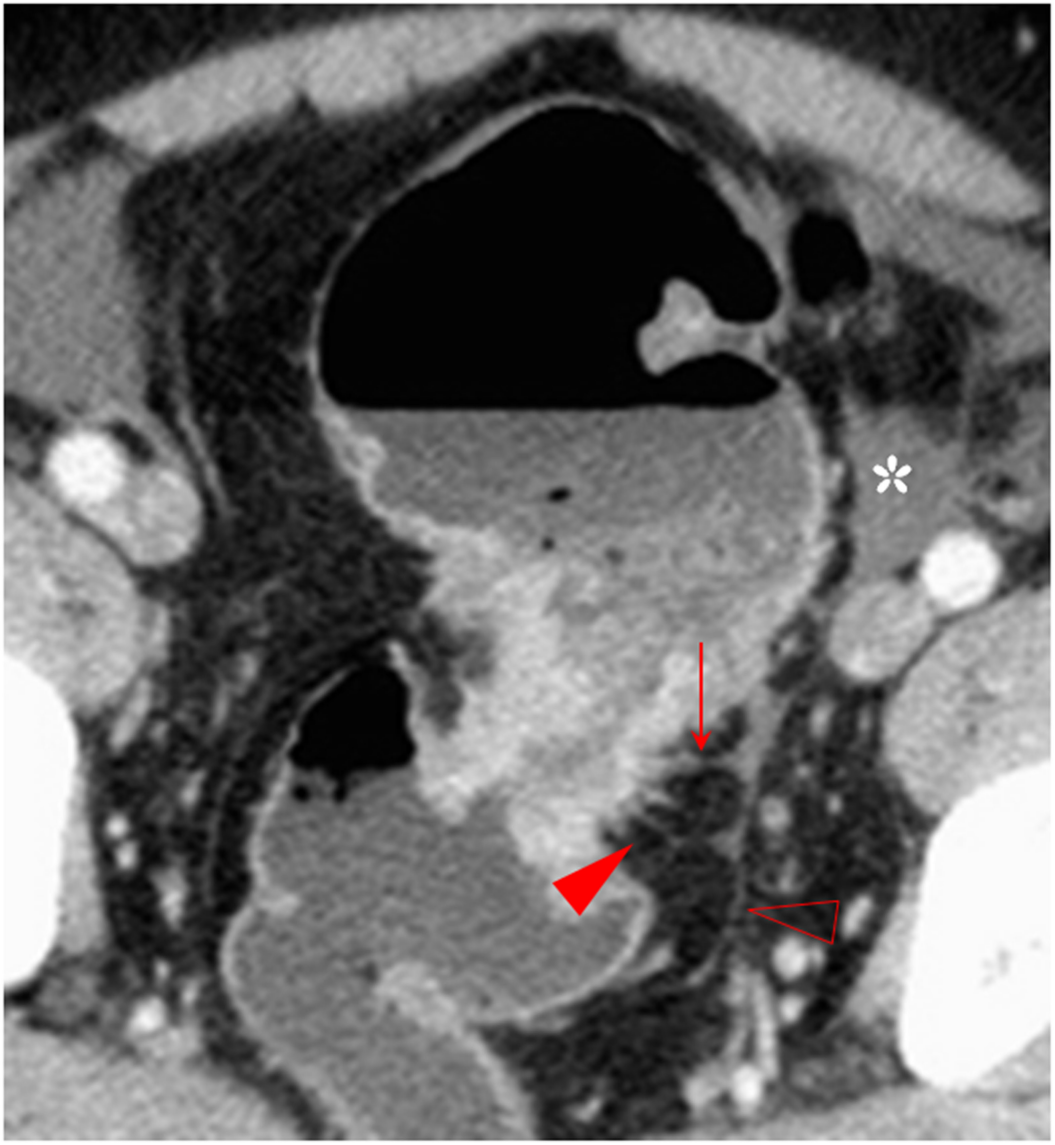
The spine sign. The transverse section of a distal sigmoid colon adenocarcinoma, pT3N2bMx, in a 68-year-old man is shown. The spines (red arrowhead) and the longitudinal long spine (hollow arrowhead) are shown. The spines appear as little spikes from the serosal side of the tumor. However, the longitudinal long spine has a long linear pattern and is away from the tumor, has a smooth serosal surface, and can kiss the spines on the tumor (solid arrow). Small amount of ascites is also present (asterisk).

**Figure 4:**
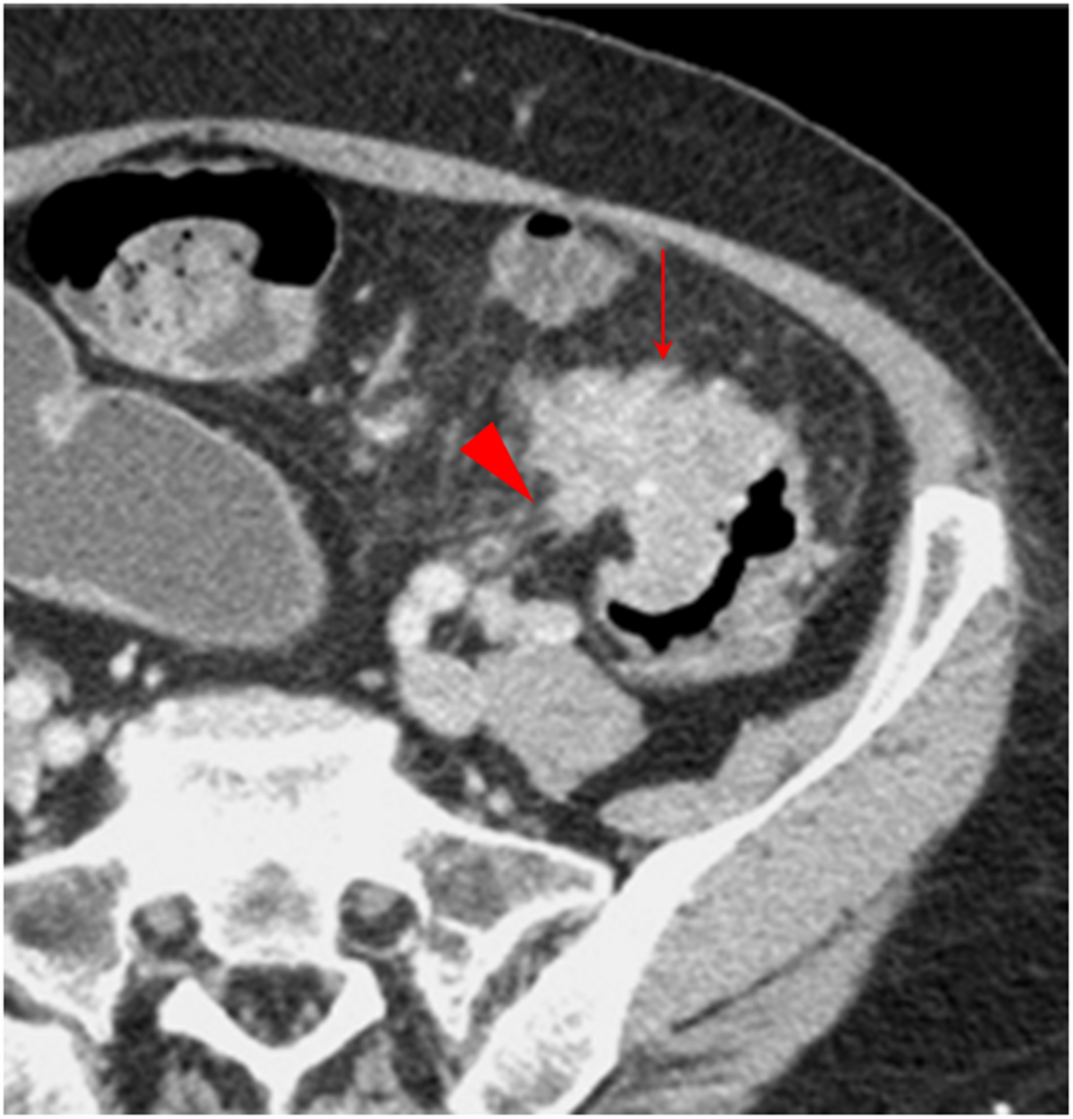
Small protuberance sign. The transverse section of a middle sigmoid colon adenocarcinoma, pT4N2aMx, in a 68-year-old woman is shown. Small protuberances (solid arrow) are shown along the serosal side outline of the tumor. Some spines are present on a small protuberance (arrowhead).

**Figure 5:**
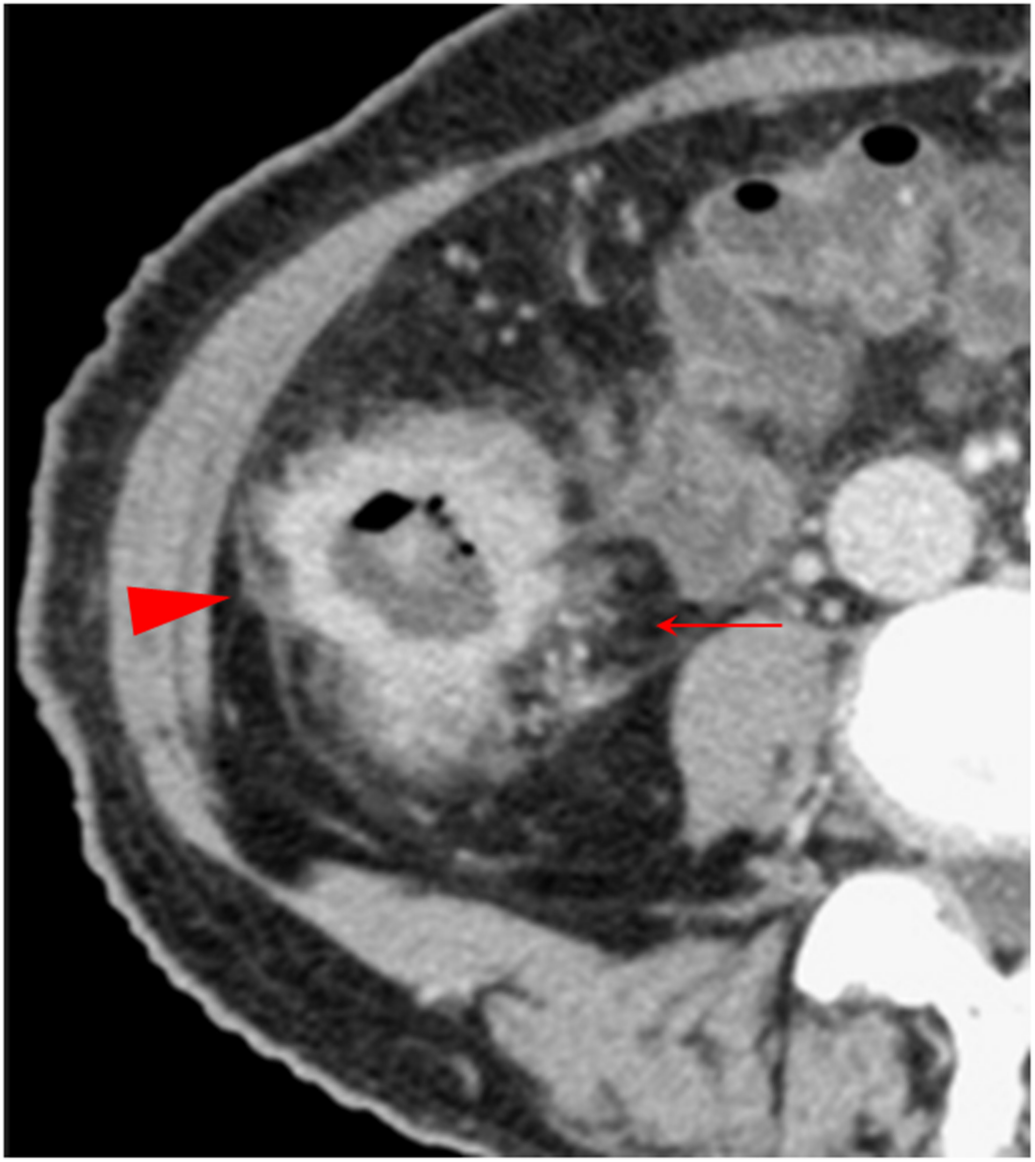
Combination of multiple CT signs. The transverse section of a middle ascending colon adenocarcinoma, pT4bN1Mx, in a 73-year-old woman is shown. The extensive reticulonodular fat stranding (solid arrow), fascia thickening, the fascia adhesion and concave, extra-fascial nodules, and streaks (arrowhead) are shown. The pericolic fat and the extra-fascial fat exhibit diverse increased attenuation patterns. Thickened lateral conal fascia with adhesion and a concave contour toward tumor is shown.

Our analysis focused on the following key features on CT images: (1) the pliability of the colon wall (soft or stiff); (2) the outline contour of the colon serosa (smooth, spinous, small or large protuberance-like); (3) presence or absence of pericolic fat stranding (local or extensively reticulonodular); (4) pericolic vascularity (increased or normal); (5) presence or absence of cancer lesion fusion with adjacent organs; (6) presence or absence of ascites; (7) tumor size (maximum diameter in mm); (8) the distance between the outer outline of the normal ascending/descending colon or cancer therein and the retroperitoneal fascia (RPF) (≤1 mm or >1 mm) (23, 24); and (9) abnormalities of the RPF adjacent to the cancers of ascending/descending colon. Definitions of the key CT features are listed in **Table 1**. CT images demonstrating typical tumor features are shown in **Figures 1**–**5**. The number and type of tumor features in different stages of colon cancer identified on CT images are presented in **Tables 2**–**4**.

**Table 1:** Definitions of the key CT features.

Features	Definitions
1. Soft or stiff	Soft: the serosal side outline of cancer lesion has the same pliability as the normal colon, i.e., showing natural, compliant, and smooth outline, and is expandable. Stiff: part or all of the serosal side outlines of cancer lesion cannot be expanded and is concave toward the luminal side. The maximum external diameter of the diseased colon is smaller than that of the normal colon.
2. Spines	Small spikes on the serosal side of cancers. The tips of the spines point toward the serosal side. Longitudinal long spine: a special type of spine, showing a linear pattern and is away from the lesion. The longitudinal long spine is parallel to the longitudinal axis of the colon with a smooth serosal surface.
3. Small protuberances	Small irregular nodular protrusions with or without small irregular wavy contour on the serosal side outline of cancer lesion.
4. Large protuberances	Single or multiple protuberances with a height ≥10 mm.
5. Smooth serosal side outline	No spine, no protuberance of any sizes, on any segment of the serosal side outline of cancer lesion.
6. Pericolic fat stranding	Abnormal soft tissue attenuation in any part of the pericolic fat. A local, amorphous, and cloud-like abnormity is referred to as the local increased attenuation or the local fat stranding. An extensive, nodule-like reticular abnormity is referred to as the extensive reticulonodular increased attenuation or the extensive reticulonodular fat stranding.
7. Increased pericolic vascularity	Increase in the number and size of the pericolic blood vessels. These vessels may be tortuous in shape.
8. Fusion with adjacent organs	The fat septum between the serosal side of cancer lesion and the adjacent organs disappears. The tumor-invaded portion of the organ shows the same attenuation as the tumor.
9. A distance between the outer outline of the normal ascending/descending colon or cancer therein and the retroperitoneal fascia	The distance (mm) is measured on the axial images. Interference of the partial volume effects should be minimized when measuring.
10. Abnormities of the retroperitoneal fascia adjacent to ascending/descending colon cancer	The abnormities are classified into three types: (1) local fascia thickening: the thickening of the fascia is restricted to a small area and no other abnormalities can be identified; (2) fascia adhesion and concave: various increased attenuation patterns connecting the fascia and the lesion, and the fascia is concave toward the lesion, in addition to the local fascia thickening; and (3) the extra-fascial nodules and streaks: abnormal nodules and streaks seen on the outer surface of the fascia.

**Table 2:** Number of tumors with diverse CT features in pT1~pT4 stages.

pT stage	No. of lesions	Colon wall		Abnormalities of serosal side outline			Pericolic fat stranding		Increased vascularity^b^	Fusion with adjacent organs ^c^	Ascites ^c^	Tumor maximum diameter ( $\bar{x}$ ± s, mm) ^d^
		Soft ^a^	Stiff ^a^	Spines	Small protuberances ^b^	Large protuberances ^c^	Local	Extensive reticulonodular ^c^				
T1	5	5	–	–	–	–	–	–		–	–	19.3 ± 5.4
T2	7	–	7	5	2	–	–		5	–	–	38.2 ± 4.2
T3	96	–	96	84	96	8	38	8	94	9	6	44.7 ± 1.5
T4	28	–	28	28	28	10	14	12	28	13	13	60.8 ± 4.4

a Soft or stiff colon wall is a significant CT feature differentiating pT1 vs. pT2~pT4 tumors (*p* < 0.001).

b Small protuberance and increased vascularity are significant CT features differentiating pT2 vs. pT3~pT4 tumors (*p* < 0.001 and *p* < 0.05, respectively).

c Large protuberances, extensive reticulonodular fat stranding, fusion with adjacent organs, and ascites are significant CT features differentiating pT3 vs. pT4 tumors (*x* ^2^ = 10.983, *p* < 0.001; *x* ^2^ = 16.633, *p* < 0.001; *x* ^2^ = 17.933, *p* < 0.001; and *x* ^2^ = 23.963, *p* < 0.001, respectively).

d Tumor sizes are significantly different between pT1~pT4 stages (F = 451.43, *p* < 0.001).

**Table 3:** Number of tumors with multiple CT features in pT1~pT4 cancers.

pT stage	No. of lesions	Spines + small protuberances ^a^	Large protuberances + ascites ^b^	Extensive reticulonodular fat stranding + ascites ^b^	Fusion with adjacent organs + ascites ^b^	Extensive reticulonodular fat stranding + fusion with adjacent organs + ascites ^b^
T1	5	–	–	–	–	–
T2	7	2	–	–	–	–
T3	96	84	2	–	–	–
T4	28	28	4	6	8	4

a Spines plus small protuberances are a significant CT feature differentiating pT2 vs. pT3~pT4 tumors (*p* < 0.001).

b Large protuberances plus ascites, extensive reticulonodular fat stranding plus ascites, fusion with adjacent organs plus ascites, and extensive reticulonodular fat stranding plus fusion with adjacent organs plus ascites are significant CT features differentiating pT3 vs. pT4 tumors (*x* ^2^ = 4.61, *p* < 0.05; *p* < 0.001; *p* < 0.001; and *p* < 0.05, respectively).

**Table 4:** Number of cases with a distance ≤1 mm or >1 mm between the outer outline of the normal ascending/descending colon or the cancers therein and RPF and the RPF abnormities.

	Normal ascending colon (106 sides)	Normal descending colon (114 sides)	Ascending colon cancer*		Descending colon cancer			
			pT3 (16 lesions^#^)	pT4 (6 lesions)	pT1 (1 lesion)	pT2 (1 lesion)	pT3 (14 lesions)	pT4 (1 lesion)
Distance to RPF ^a^								
≤1 mm	102	112	16	6	1	1	14	1
>1 mm	4	2	–	–	–	–	–	–
CT features of RPF								
Normal	106	114	–	–	1	1	2	–
Local thickening	–	–	16	6	–	–	12	1
Adhesion and concave	–	–	9	5	–	–	5	1
Extra-fascial nodules and streaks ^b^	–	–	2	5	–	–	1	1
Adhesion and concave + extra-fascial nodules and streaks ^b^	–	–	2	5	–	–	1	1

*A case of pT4 ascending colon cancer was not included because of its malrotation.

^#^Two lesions were not included: one was not in the naked area, and the RPF of the other one could not be clearly shown due to very thin body and the dilated intestine.

RPF, retroperitoneal fascia.

a Normal ascending/descending colons vs. cancers therein; the incidence of distance ≤1 mm was not significant different (*p* > 0.05).

b Extra-fascial nodules and streaks, and adhesion and concave plus extra-fascial nodules and streaks are significant CT features differentiating pT3 vs. pT4 tumors (*p* < 0.001 and *p* < 0.001, respectively).

The occurrence rate of each CT feature varied across different tumor stages. Abnormal colon wall pliability (i.e., reduced softness or increased stiffness of the colon wall) was significantly more common in pT2~pT4 tumors than in pT1 tumors (*p* < 0.001) (**Table 2**), making the colon wall pliability a marked feature for differentiating the colon cancers of pT2~pT4 stages from those in the pT1 stage. Small protuberances, increased vascularity, and presence of small protuberances plus spines were strikingly more common in pT3~pT4 tumors than in pT2 tumors (*p* < 0.001, *p* < 0.05, and *p* < 0.001, respectively) (**Table 2**). The small protuberances in pT2 cancers were generally fewer in number and smaller in size, whereas, in the pT3–pT4 cancers, they were generally more abundant with variable sizes.

The CT features that can differentiate the pT3 and pT4 tumors included large protuberances (*x* ^2^ = 10.98, *p* < 0.001), extensive reticulonodular fat stranding (*x* ^2^ = 16.63, *p <* 0.001), fusion with adjacent organs (*x* ^2^ = 17.93, *p* < 0.001), and ascites (*x* ^2^= 23.96, *p* < 0.001), all significantly more common in pT4 tumors (**Table 2**). In addition, multi-features were more common in pT4 tumors than in pT3 tumors, including large protuberances plus ascites (*p* < 0.05), extensive reticulonodular fat stranding plus ascites (*p* < 0.001), fusion with adjacent organs plus ascites (*p* < 0.001), pericolic fat with extensive reticulonodular fat stranding plus fusion with adjacent organs plus ascites (*p <* 0.05) (**Table 3**), extra-fascial nodules and streaks (*p* < 0.001), and adhesion and concave plus extra-fascial nodules and streaks (*p* < 0.001) (**Table 4**).

**Figure 6:**
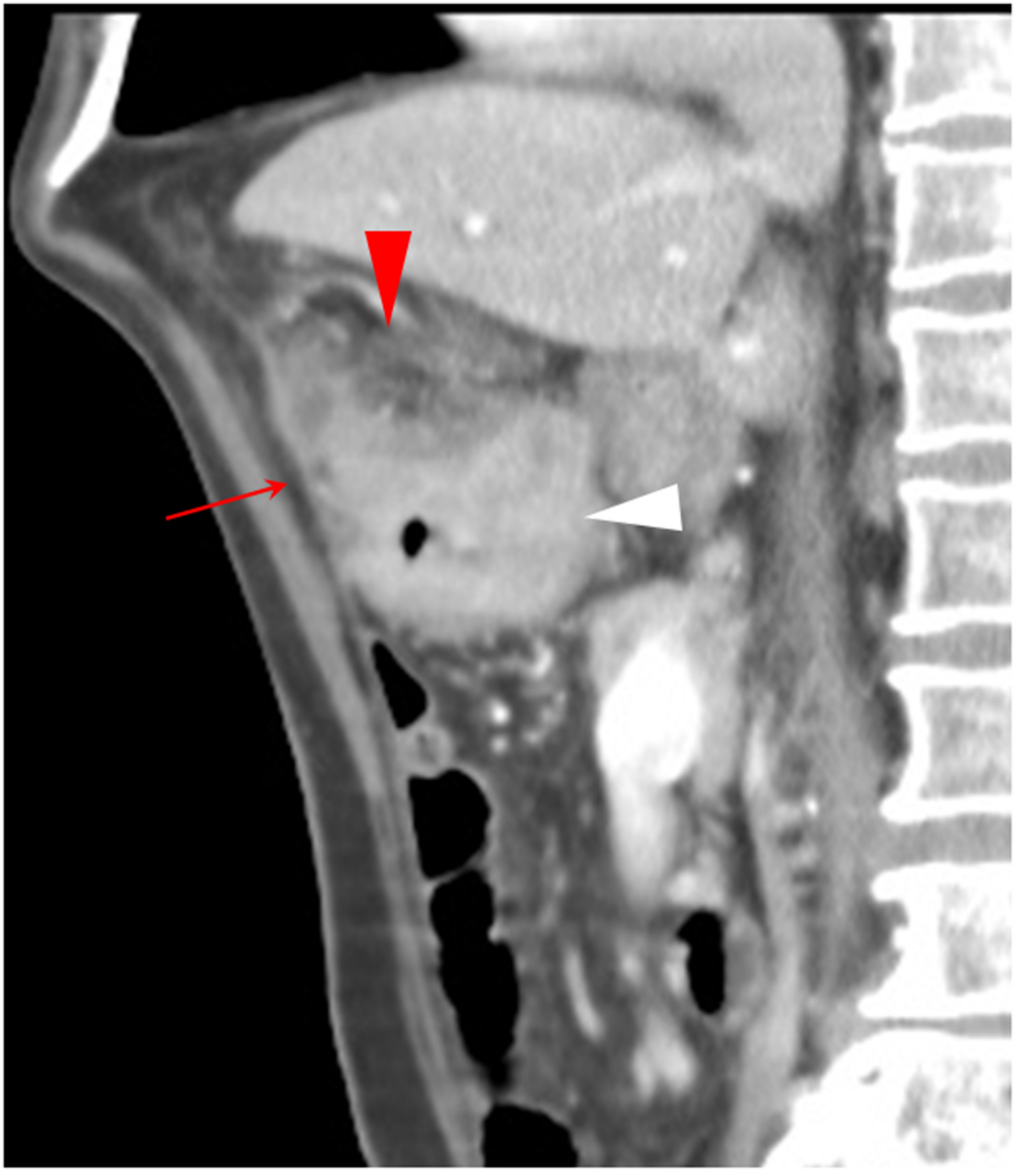
The sagittal section of a transverse colon adenocarcinoma (white arrowhead), pT3N0Mx, in an 80-year-old man is shown. Extra-peritoneal adipose tissue is clearly visible (solid arrow). An extensive, irregular, and heterogeneous soft tissue attenuation pattern on the tumor and adjacent to the anterior parietal peritoneum is shown (red arrowhead).

In 11 out of the 96 pT3 cancers and 3 out of the 28 pT4 cancers, we also found an additional CT feature: an extensive, irregular, and heterogeneous soft tissue attenuation mass surrounding the lesions and adjacent to the parietal peritoneum. The extra-peritoneal adipose tissues were clearly visible in pT3 cancers (**Figure 6**), but the fat stranding was only present in pT4 cancers. This CT feature resembles the extra-RPF adipose tissues in pT3 and pT4 cancers of the ascending/descending colons.

As shown in **Table 1**, there was a clear trend of CT features being more common in advanced stages (pT3 to pT4) and the tumor sizes increased with more advanced pT stages (F = 451.43, *p* < 0.001) (**Table 1**). In contrast, none of the above CT feature was seen in the pT1 tumors. In addition, tumors in the pT4 stage tend to exhibit more multi-features (**Tables 3**, **4**).

### 3.3 Histopathological characteristics of the cancer front (the serosal side of cancer)

To study the histopathological characteristics of the cancer front, we assessed (1) the extent and distribution of DR and IR at the tumor front (i.e., the cancer serosal side); (2) the nature of the abnormal tissues between the serosal surface of muscularis propria and serosa; and (3) possible abnormalities beyond serosa.

Except for intra-mucosal cancers, cancers of all pT stages showed DR and IR of various extents at the cancer front. However, compared to DR, IR only affected smaller anatomical area. As shown in previous studies, IR was mainly composed of inflammatory cells with lymphocytes being the most predominant cell type (this condition is referred to as “follicular inflammatory reaction” (17)) and was generally surrounded by DR. Tumors of the pT1 stage did not show any abnormalities in muscularis propria and sub-serosal fat, whereas tumors of the pT2 stage exhibited simple DR and IR in the sub-serosal fat. In contrast, the tumors of the pT3 and pT4 stages exhibited cancer foci enwrapped by the DR in the sub-serosal fat and beyond the serosa, respectively, in addition to simple DR and IR.

### 3.4 Histopathological interpretations of the CT features

**Figure 7:**
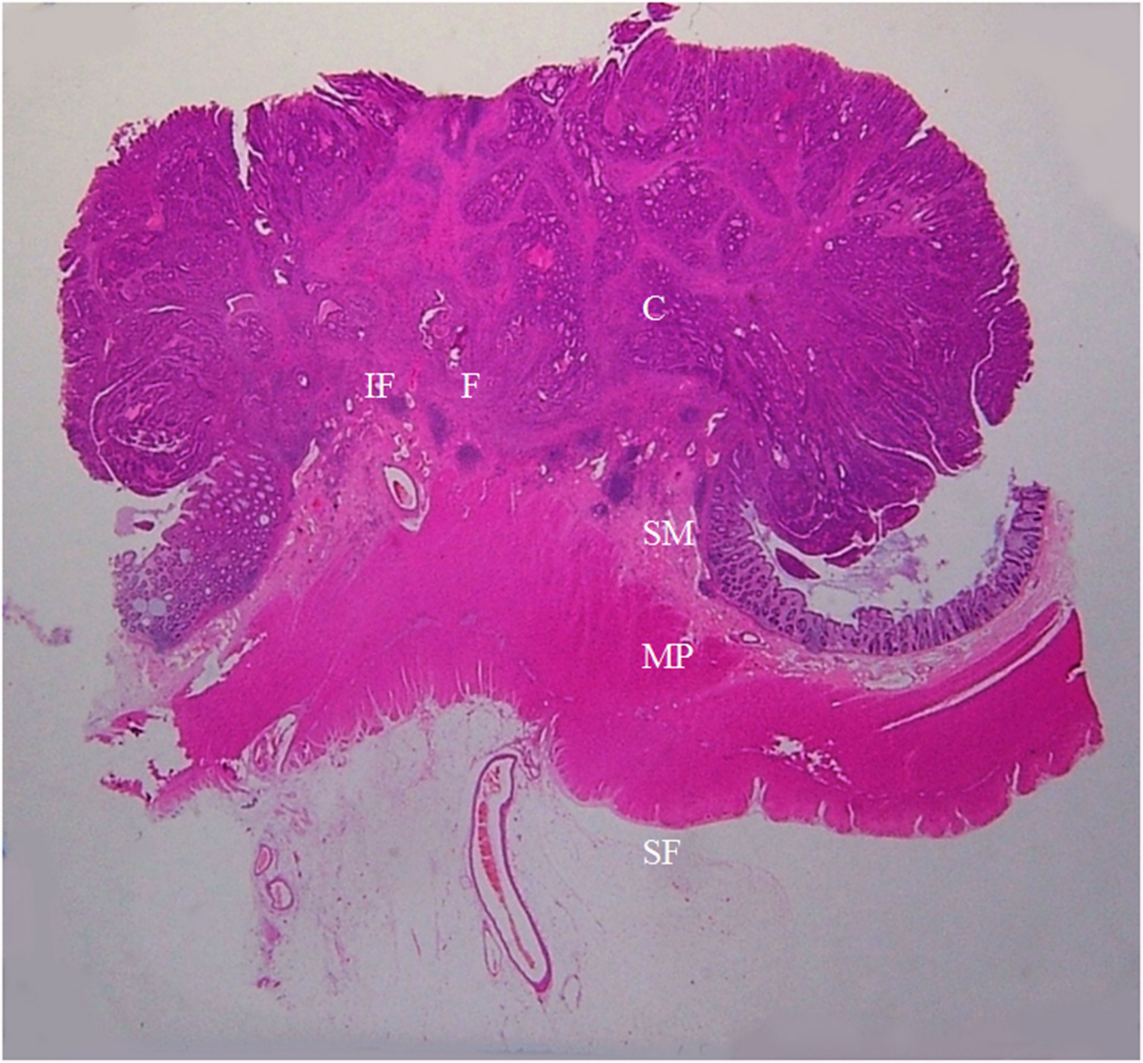
A section of the proximal descending colon adenocarcinoma, pT1N0Mx, in a 42-year-old woman is shown (H&E stain, original magnification ×1). Invasion of cancer cells (C) to submucosa (SM) is visible. A large amount of fibrous tissue (F) and a few inflammatory foci (IF) are present at the cancer front. The muscularis propria (MP) and serosal fat layer (SF) are normal. On CT scan, this lesion appears smooth and soft on the serosal side outline.

**Figure 8:**
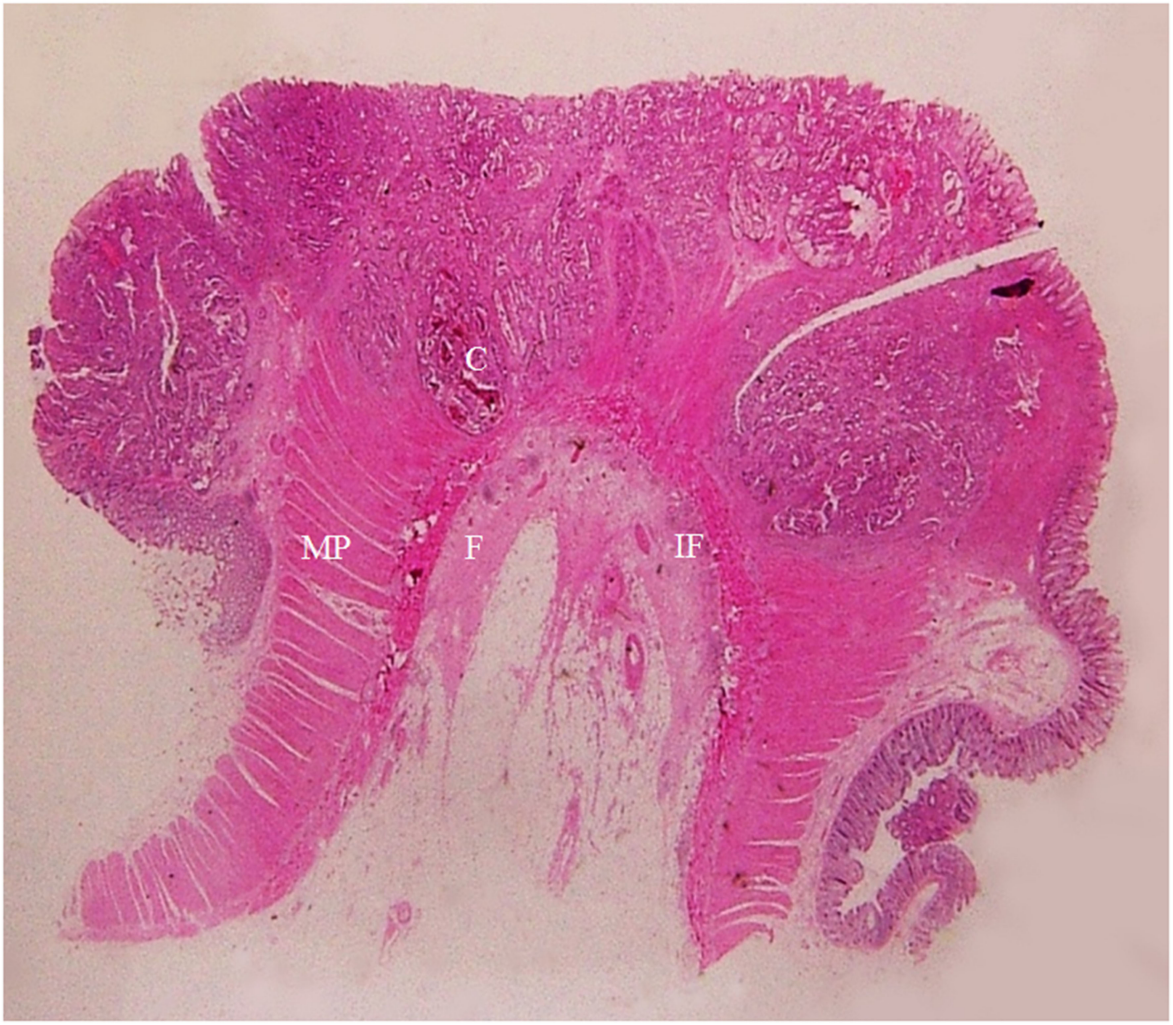
A section of a middle sigmoid colon adenocarcinoma, pT2N1cMx, in an 80-year-old man is shown (H&E stain, original magnification ×1). Invasion of cancer cells (C) to muscularis propria (MP) is visible. A large amount of fibrous tissue (F) and a few inflammatory foci (IF) at the surface of muscularis propria are shown. The fibrous tissue extends toward serosal side with a spike-like pattern. On CT scan, this lesion shows a stiff serosal side outline accompanied by spines.

**Figure 9:**
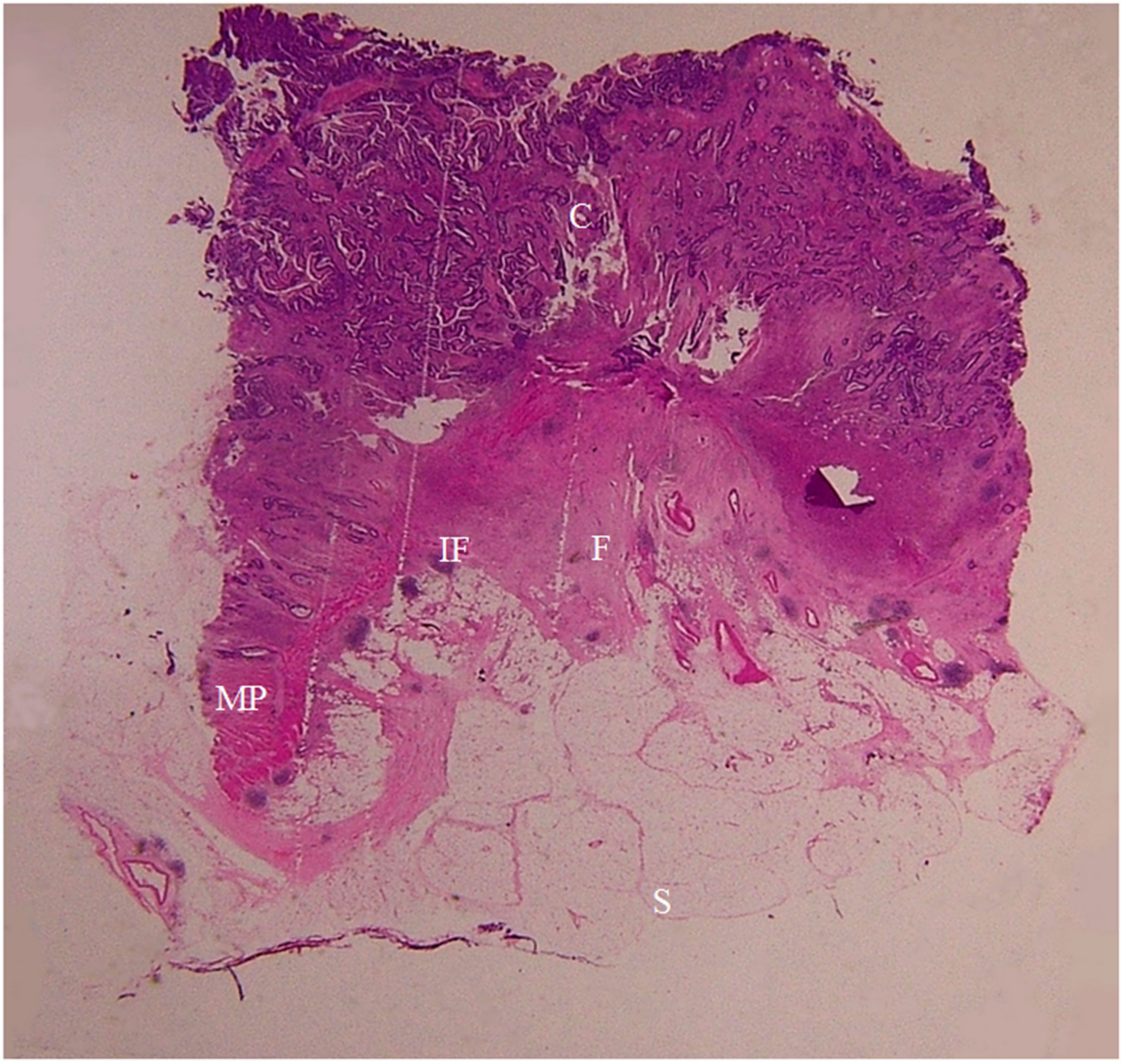
A section of a proximal descending colon adenocarcinoma, pT2N0Mx, in a 37-year-old woman is shown (H&E stain, original magnification ×1). Invasion of cancer cells (C) to the muscularis propria (MP) is visible. A large amount of fibrous tissue (F) and a few inflammatory foci (IF) are present at the surface of muscularis propria. The fibrous tissue extends toward the serosal side with diverse patterns (spike-like, mound-like, aristae-like, or irregular streaks). The serosa (S) is intact. CT scan shows a stiff serosal side outline accompanied by spines and small protuberances with clear pericolic fat.

**Figure 10:**
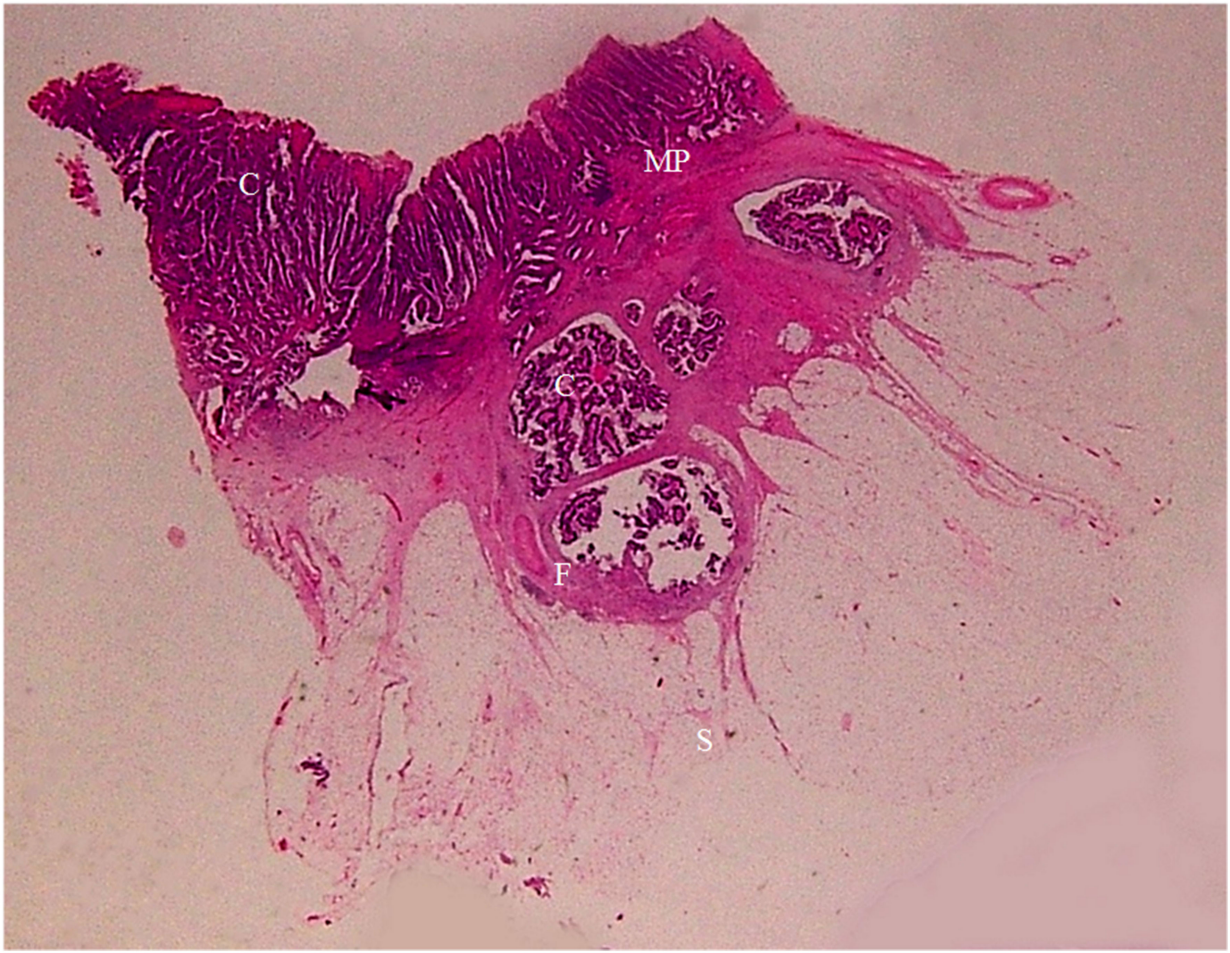
A section of a middle sigmoid colon adenocarcinoma, pT3N0Mx, in a 67-year-old woman is shown (H&E stain, original magnification ×1). Cancer cells (C) have invaded through the muscularis propria (MP) into the sub-serosal fat layer. A large amount of fibrous tissue (F) at the cancer front enwraps the cancer foci and extends toward serosal side in a spike-like shape. Serosa (S) is intact. On CT scan, this lesion shows stiff serosal side outline accompanied with spines and small protuberances.

**Figure 11:**
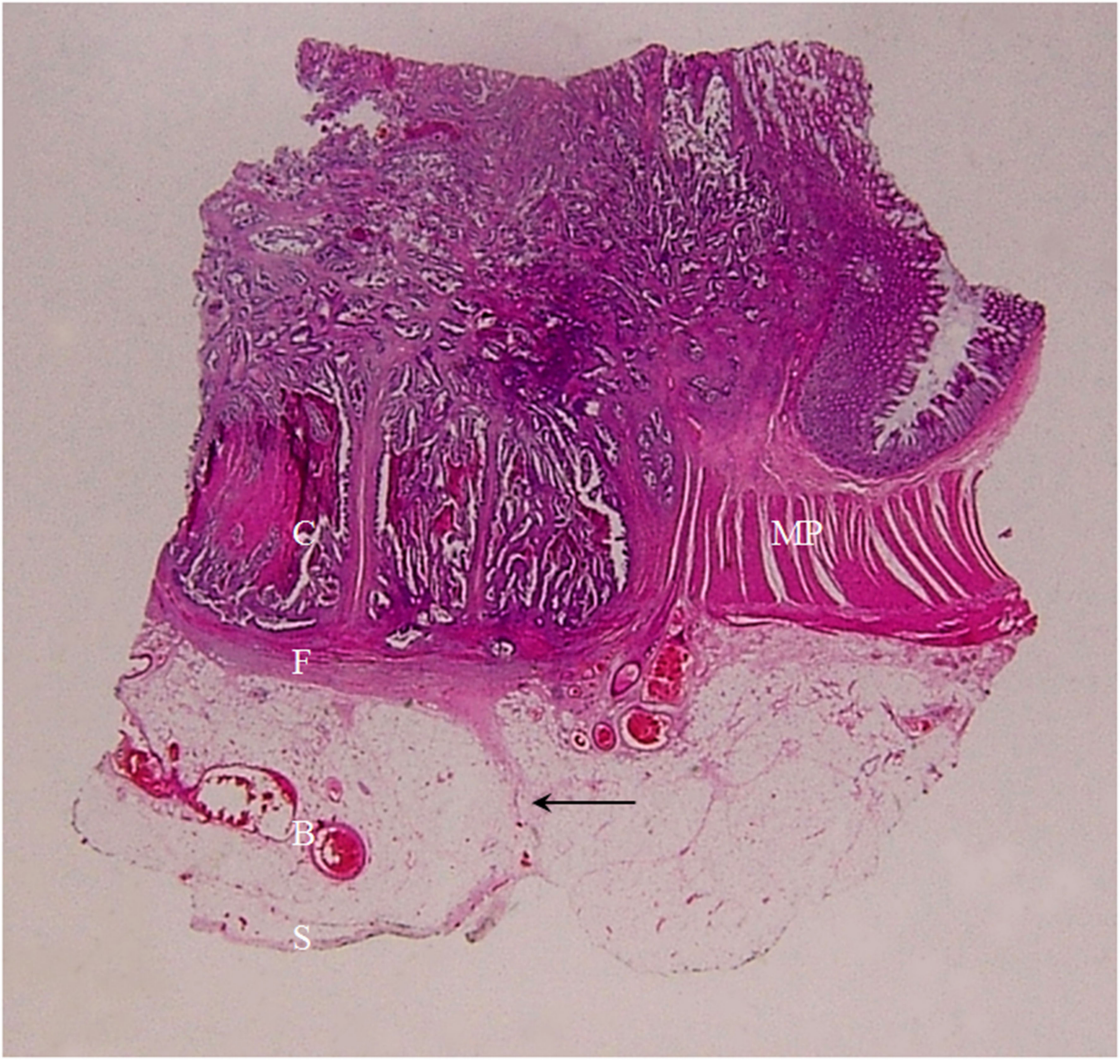
A section of a middle sigmoid colon adenocarcinoma, pT3N0Mx, in a 73-year-old woman is shown (H&E stain, original magnification ×1). Cancer cells (C) have invaded through the muscularis propria (MP) into the sub-serosal fat layer. A large amount of fibrous tissue is present at the cancer front, some giving rise to a “lamina-like” structure with a smooth outside surface (F). A thin layer of fibrous tissue underneath the serosal epithelial cells (S) touching on the spines on the muscularis propria (solid arrow). Increased vascularity (multiple blood vessels with large diameter) in the sub-serosal fat layer can be seen (B). On CT scan, this lesion exhibits stiff but relatively smooth serosal side outline, with increased vascularity, longitudinal long spines and the “kissing-like” spines.

**Figure 12:**
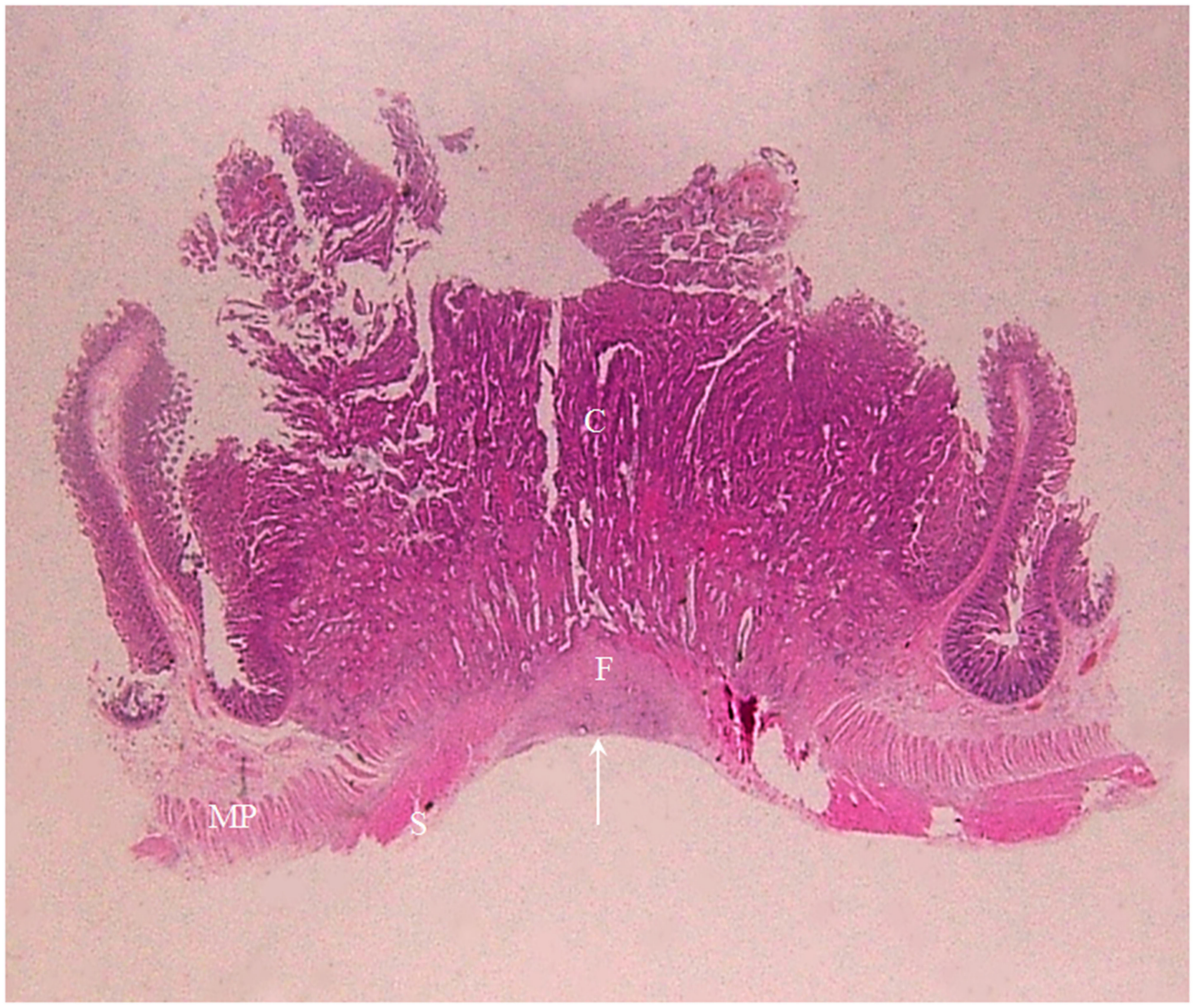
A section of a middle transverse colon adenocarcinoma, pT4bN1cMx, in a 79-year-old woman is shown (H&E stain, original magnification ×1). Cancer cells (C) have invaded through the muscularis propria (MP) and the serosal epithelial cells (S). Numerous cancer foci are present in the fibrous tissue (F) located in the serosal layer with a smooth serosal outline (white arrow). This lesion exhibits a stiff but relatively smooth serosal side outline on CT.

We analyzed the Hematoxylin-eosin staining (H&E) slides of the resected tissues to seek a histopathological interpretation of the imaging features seen on CT scans. We found that the observed CT features at the serosal side of colon cancer mostly represented the pure DR and/or cancer foci enwrapped by DR in the sub- and/or extra-serosal fat layers. These histopathological components showed a soft tissue attenuation on CT scan (i.e., showing a positive CT value) and were in sharp contrast with the surrounding fat (i.e., showing a negative CT value). The imaging features on CT scan were unlikely to be the result of IR as the number and size of IR foci were negligible (**Figures 7**–**12**).

#### 3.4.1 Clinical and pathological relevance of the pliability of the colon wall on CT scan

The soft colon wall feature on CT scan translates to the absence of cancer cells, DR and IR in the muscularis propria and the serosal layer (**Figure 7**). This feature was only present in the pT1 tumors. The stiff colon wall feature, on the other hand, indicates the presence of cancer cells and striking DR in lamina propria, even the serosal layer or beyond serosa. Tumors with this stiff colon wall feature were all in pT2~pT4 stages. The occurrence rate of the soft and stiff colon wall signs between the pT1 tumors (n = 5) and pT2~pT4 tumors (n = 131) was significantly different (*p* < 0.001). The PPV for soft colon wall sign in diagnosing the pT1 tumors and the PPV for stiff colon wall sign in diagnosing the pT2~pT4 tumors all reached 100% with a 100% sensitivity (**Table 5**).

**Table 5:** Diagnostic performance of the soft colon wall for pT1 cancers.

CT features	Sensitivity (%)	Specificity (%)	Positive predictive value (%)	Negative predictive value (%)	Positive likelihood ratio (multiples)	Negative likelihood ratio (multiples)
Soft wall	100	0	100	0	1	0

#### 3.4.2 Clinical and pathological relevance of the outline contour of the colon wall and other features on CT scans

Tumors in the pT1 stage generally had no remarkable features on CT scan; thus, CT features like spine and small protuberance are not present, and the pT1 tumors have smooth contour on the serosal side. On the other hand, all tumors in pT2~pT4 stages showed spine, small or large protuberances. Histologically, the tumors in the pT1 stage had no cancer cells and DR in muscularis propria and sub-serosal fat (**Figure 7**).

The spines seen in the pT2 cancers represented the pure DR between surface of lamina propria and serosa (**Figures 8**, **9**), and the spines of pT3~pT4 tumors represented the DR of the cancer front in the serosal layer or beyond serosa (**Figure 10**).

The longitudinal long spines that were only detectable in pT3 cancers represented the thin layer of pure DR underneath the serosal epithelial cells, and this special type of spines may intercalate the DR on the lamina propria side (**Figure 11**).

The small protuberances of pT2 cancer represented the pure DR on the surface of lamina propria (**Figure 9**). The small protuberances of pT3~pT4 cancers represented the sub-serosal or extra-serosal cancer cell foci enwrapped by DR (**Figure 10**).

The large protuberances were detected only in pT3 and pT4 cancers, and they represented farther and deeper infiltration of cancer cells toward the serosal side.

Several CT features showed significant diagnostic value for colon cancer. For example, the “small protuberances” sign showed a PPV of 98.4% for the pT3~pT4 tumors with a sensitivity of 100% and a specificity of 71.4%. The “spines plus small protuberances” signs showed a PPV of 98.2% for the pT3~pT4 tumors with a sensitivity of 90.3% and a specificity of 71.4% (**Table 6**).

**Table 6:** Diagnostic performance of the small protuberances, vascularity, and spines plus the small protuberances for pT3~pT4 cancers.

CT features	Sensitivity (%)	Specificity (%)	Positive predictive value (%)	Negative predictive value (%)	Positive likelihood ratio (multiples)	Negative likelihood ratio (multiples)
Small protuberances	100	71.4	98.4	100	3.5	0
Increased vascularity	98.3	28.6	96.1	50	1.38	0.06
Spines + small protuberances	90.3	71.4	98.2	29.4	3.16	0.14

The diagnostic performance of the statistically significant CT features (**Table 7**) for pT4 cancers varied in terms of sensitivity, specificity, PPV, and negative predictive value. The low sensitivity of these parameters for the pT4 tumors was likely due to lower occurrence rate of these CT features.

**Table 7:** Diagnostic performance of the eight group of CT features for pT4 cancers.

CT features	Sensitivity (%)	Specificity (%)	Positive predictive value (%)	Negative predictive value (%)	Positive likelihood ratio (multiples)	Negative likelihood ratio (multiples)
Large protuberances	35.7	91.7	55.6	83.0	4.3	0.7
Extensive reticulonodular fat stranding	42.9	91.7	60	84.6	5.1	0.6
Fusion with adjacent organs	46.4	90.6	59.1	85.3	5.0	0.6
Ascites	46.4	93.8	68.4	85.7	7.4	0.6
Large protuberances plus ascites	14.3	97.9	66.7	79.7	6.9	0.9
Extensive reticulonodular fat stranding + ascites	21.4	100	100	81.4	–	0.8
Fusion with adjacent organs + ascites	28.6	100	100	82.8	–	0.7
Extensive reticulonodular fat stranding + fusion with adjacent organs + ascites	14.3	100	100	80	–	0.9

In some pT2~pT4 tumors, the entire or partial serosal contour was smooth. Histologically, the smooth contour represented the smooth serosal surface of pure DR and/or DR enwrapping cancer cells that were in parallel with lamina propria (**Figures 11**, **12**). In pT2 tumors, the smooth contour may also represent the normal serosal surface of muscularis propria.

Pericolic fat stranding on CT images represented the pure DR and/or cancer cells enwrapped by DR that accumulated in the sub-serosal fat layer or extra-serosal fat. The pericolic fat stranding sign might not be detectable if the DR and/or cancer cells enwrapped by DR were sparse (**Figure 9**). On the other hand, if these tissues were disorderly scattered in the fat and the fat was significantly reduced, then the extensive reticulonodular pattern would appear on CT scan.

The mucinous adenocarcinoma of pT4 stage also showed extensive reticulonodular pattern, which histopathologically represented mucinous carcinoma foci separated by DR.

Increased vascularity on the CT scan represented an increase in the number and size (diameter) of the blood vessels next to tumor tissues at the histopathological level (**Figure 11**).

The fusion of cancer lesion with adjacent organs on CT images represented different histopathological conditions in pT3 and pT4 tumors, respectively. The fusion of pT4 cancers with adjacent organs reflects the infiltration of cancer cells and DR into the neighboring organs, whereas, in the pT3 cancers, these CT features only represent the adhesion of the massive DR.

#### 3.4.3 The relevance of the distance between the outer outline of the normal ascending/descending colon or the tumors therein and retroperitoneal fascia

As shown in the **Table 4**, the occurrence rate of the distance between the outer outline of the normal ascending/descending colon (n = 220) and the cancers therein (n = 39) with RPF being ≤1 mm did not show any statistically significant difference (*p* > 0.05).

#### 3.4.4 Clinical and pathological relevance of the abnormal retroperitoneal fascia between the pT3 and pT4 ascending/descending colon cancers

The CT sign of extra-fascial nodules and streaks adjacent to ascending/descending colon cancer represented the pure DR at pT3 cancers front, or cancer cell foci enwrapped by DR at pT4 cancers front, respectively. As shown in **Table 4**, between the pT3 (n = 30) and pT4 (n = 7) ascending/descending colon cancers, a significant difference in the occurrence rates of the extra-fascial nodules and streaks (*p* < 0.001) and the adhesion and concave plus extra-fascial nodules and streaks (*p* < 0.001) was observed. The presence of the extra-fascial nodules and streaks was closely accompanied by the adhesion and concave.

As shown in **Table 8**, the extra-fascial nodules and streaks were diagnostic of the pT4 ascending/descending colon cancers, with a sensitivity of 85.7%, a specificity of 90%, a PPV of 66.7%, a negative predictive value of 96.4%, a positive likelihood ratio (+LR) of 8.6 times, and a negative likelihood ratio (−LR) of 0.2 times, respectively.

**Table 8:** Diagnostic performance of the retroperitoneal fascia abnormalities for pT4 ascending/descending colon cancers.

CT features	Sensitivity (%)	Specificity (%)	Positive predictive value (%)	Negative predictive value (%)	Positive likelihood ratio (multiples)	Negative likelihood ratio (multiples)
Extra-fascial nodules and streaks	85.7	90	66.7	96.4	8.6	0.2
Adhesion and concave + extra-fascial nodules and streaks	85.7	90	66.7	96.4	8.6	0.2

## 4 Discussion

Accurate pre-operative diagnosis and pre-treatment clinical T staging are crucial components of the wholistic management of patients with colon cancer (1–4, 25). Such an unmet clinical need cannot be achieved by postoperative pathological tumor staging (pT), as this approach essentially reflects the stepwise infiltration and penetration of cancers cells from the mucosa toward submucosa, muscularis propria, serosa, and, finally, adjacent organs.

The standard method for pre-operative clinical T staging is by CT. However, clearly distinguishing the multi-layered colon tissues, identifying the cancer cell infiltration or penetration into the colon wall, and revealing the presence of DR remain critical challenges, often leading to a low accuracy of clinical T staging in patients with colon cancer (10–12). So far, no CT reference standards have been established for the clinical T staging for colon cancer (6, 7).

In this study, we aimed to corroborate the CT features of the serosal side of colon cancer with histopathological findings. We focused on the significantly different CT features between the colon cancers of different stages.

In cancers of the pT1 stage, soft colon wall was the only features seen on CT scan, whereas cancers of the pT2~pT4 stages all had “stiff wall” sign. The “soft wall sign” is a highly sensitive and specific sign to differentiate the colon cancers of the pT1 stage and those in other stages, with sensitivity and PPV reaching 100%. The “stiff colon wall” sign reflects the involvement of cancer cells with DR at its front in the muscularis propria, and thus indicates more advanced tumor stage. On the other hand, the presence of the obvious DR in submucosa of pT1 cancers does not affect the pliability of the colon wall; hence, no “stiff colon wall” sign may be seen on CT scan as muscularis propria and sub-serosal fat of pT1 cancer remain intact. Therefore, the colon wall pliability is the characteristic CT feature that can be used to differentiate the tumors of the pT1 stages from those of the pT2~pT4 stages.

Small protuberances are a promising CT sign for differentiating the tumors of pT2 and pT3~pT4 stages. The small protuberances in pT3~pT4 cancer often represent involvement of the sub-serosal or outside serosa by cancer cells enwrapped by DR, and they tend to have a higher occurrence rate with incalculable number and variable patterns or sizes. Our studies revealed an outstanding diagnostic performance of the small protuberances for pT3~pT4 tumors, with a sensitivity and a specificity reaching 100% and 71.4%, respectively. Small protuberances in pT2 cancers reflect the pure DR on the surface of muscularis propria, and they generally have a lower occurrence rate, smaller numbers and smaller sizes. We thus recommend that small protuberances on CT be a reliable sign for differentiating the colon cancers of the pT2 stages from those of pT3~pT4 stages.

The presence of four CT features in advanced stage tumors (pT3~pT4), namely, large protuberances, extensive reticulonodular fat stranding, tumor fusion with adjacent organs, and ascites, indicates deeper and wider spread of cancer cells (e.g., to the serosa). However, the occurrence rate of each of these features is low and differs between pT3 and pT4 stages. Thus, each of these signs alone may have a relatively lower sensitivity (e.g., the sensitivity for pT4 tumors ranged from 35.7% to 46.4%) despite high specificity (e.g., specificity for pT4 tumors ranged from 90.6% to 93.8%). Combination of two or more of these four CT signs can more accurately and sensitively differentiate tumors of the pT3 from pT4 stages.

Other CT signs such as extra-fascial nodules and streaks also showed a promising diagnostic value in differentiating the pT3 from pT4 tumors of the ascending/descending colon. Our studies showed that these features may help diagnose pT4 tumors with a sensitivity of 90%, a specificity of 85.7%, and a PPV of 96.4%.

Our studies also demonstrated the value of the extra-peritoneal fat in tumor staging if the tumor is adjacent to the parietal peritoneum. Clear extra-peritoneal fat indicates pT3 tumors, whereas the presence of extra-peritoneal fat stranding often suggests the tumors are at the more advanced pT4 stage.

Previous studies by others (23, 24) indicate that the distance between the outer outline of the ascending/descending colon cancer and RPF is a marker for tumor invasion and that a distance value of ≤1 mm is indicative of a late stage tumor. Our data, however, could not confirm this.

Our study has several noteworthy limitations. First, the number of cases in the pT1, pT2, and pT4 stages is relatively small, and this can potentially lead to biased results. Second, this is a retrospective cross-sectional study, and the disagreement between the CT features and the histopathological findings in a given patient may occur. More studies in large patient cohorts from multiple centers and ideally prospective studies are needed to confirm our findings.

## 5 Conclusion

We have identified several key CT features at the serosal side of colon cancer, including colon wall pliability (being soft or stiff), small or large protuberances, extensive reticulonodular fat stranding, fusion with adjacent organs, ascites, and the extra-fascial nodules/streaks, that can be used as reliable indicators for pre-treatment clinical T staging. The findings are of great value in the wholistic management of patients with colon cancer.

## Data Availability

The original contributions presented in the study are included in the article/supplementary material. Further inquiries can be directed to the corresponding author.
